# Left hemispheric α band cerebral oscillatory changes correlate with verbal memory

**DOI:** 10.1038/s41598-020-72087-3

**Published:** 2020-09-14

**Authors:** Toshihiko Araki, Yoshiyuki Watanabe, Masayuki Hirata

**Affiliations:** 1 Department of Medical Technology, Osaka University Hospital, 2-15, Yamadaoka, Suita, Osaka 565-0871 Japan; 2 Division of Health Sciences, Osaka University Graduate School of Medicine, Suita, Osaka Japan; 3 Department of Radiology, Shiga University of Medical Science, Otsu, Shiga Japan; 4 Department of Neurological Diagnosis and Restoration, Osaka University Graduate School of Medicine, Suita, Osaka Japan

**Keywords:** Neuroscience, Neurology

## Abstract

Event-related synchronisation (ERS) and event-related desynchronisation (ERD) have been observed via magnetoencephalography (MEG) in the language-dominant hemisphere. However, the relationship between ERS/ERD and clinical language indices is unclear. Therefore, the present study evaluated brain activity utilising MEG during a verb generation task in 36 subjects and determined ERS/ERD power values in θ, α, β, low γ and high γ frequency bands. To measure clinical language indices, we adopted Wechsler Memory Scale-revised. We observed ERD in the α band from the bilateral occipital to the left central brain region, in the β band from the bilateral occipital to the left frontal region and in the low γ band a high-power signal in the left frontal region. We also observed ERS in the θ band in bilateral frontal region and in the high γ band in bilateral occipital region. Furthermore, we found a significant negative correlation between α-band ERD power at the left postcentral gyrus and medial superior frontal gyrus and verbal memory score (correlation coefficients =  − 0.574 and − 0.597, respectively). These results suggest that individuals with lower linguistic memory have less desynchronised α-band ERD power and α-band ERD power in the left hemisphere may be a neurophysiological biomarker for verbal memory.

## Introduction

In recent years, neuroimaging studies have been conducted on a whole-brain level utilising modalities such as functional magnetic resonance imaging (fMRI), positron emission tomography (PET) and magnetoencephalography (MEG). Each of these techniques exploits different facets of the brain’s physiological response and varies in the degree to which they capture the spatiotemporal dynamics of neurophysiological activity. These methods have revealed that the activities of various brain regions, such as Broca’s area and Wernicke’s region, contribute to language processing^1,2^. fMRI is based on blood oxygen-level-dependent contrast imaging and can achieve almost sub-millimetre accuracy in the spatial localisation of neuronal activity^3^. However, this relatively high spatial resolution is not matched in temporal accuracy in essence due to the relatively slow speed of hemodynamic changes in response to neuronal activity, which fundamentally limits the temporal detail which is inherently limited to a timescale of seconds^4^. PET can determine significant local changes in cerebral blood flow to compare to behavioural tasks with control states, which can identify net changes in local synaptic activity^2^. However, the disadvantages of PET include low temporal resolution even compared with that observed with fMRI and invasive radiation exposure.

MEG can directly measure the electrical activity of neurons as a weak magnetic field variation, has higher spatial resolution than electroencephalography (EEG) and has much higher temporal resolution than fMRI and PET. Therefore, it is an excellent method for measuring brain function among various modalities^5^ and providing information regarding the relative timing of electrical activity in distinct anatomical structures. Neural oscillations are a fundamental mechanism for enabling coordinated neural activity during brain functioning^6,7^. Responses measured using MEG include event-related synchronisation (ERS), in which the power at a specific frequency band increases, and event-related desynchronisation (ERD), in which the power decreases after a stimulus^8^. It has been shown that cerebral oscillatory changes such as ERS and ERD are related not only to basic brain functions^9,10^ such as movements and sensations, but also higher brain processing function such as memory^11,12^ and language functions^13,14^.

It has been reported that the spatiotemporal distribution of ERS/ERD varies in each frequency band in healthy subjects during a silent reading task^15^. It has also been reported that cerebral oscillatory changes in the α and β bands integrate word meaning and syntactic information, while those in the γ band play a role in predicting the next word during sentence processing^16,17^. Thus, it is conceivable that ERS/ERD in each frequency band may have differential language processing functions. Furthermore, it has been reported that lateralising variations in ERD in the β band or the low γ band can function as a non-invasive index for estimating the language-dominant hemisphere during language tasks in patients with epilepsy and brain tumour^18–20^. Thus, cerebral oscillatory variations in MEG signal may be related to language function when detected in the context of a language-related task. However, the relationship between brain activity measured by MEG and actual linguistic ability evaluated by neuropsychological tests remains to be elucidated and a method for evaluating linguistic ability using MEG has not been established to date. Previous studies using modalities other than MEG have clarified the relationship between the index obtained by neuroimaging and the index of actual cognitive function. For healthy adults, it has been reported that memory score correlates with the brain volume of the hippocampus as calculated by MRI^21^. In healthy elderly subjects, it has been reported that single-photon emission computed tomography shows a negative correlation between the reduction of regional cerebral blood flow in the left inferior frontal gyrus and memory performance^22^. Based on the results of these studies, we hypothesised that there is some correlation between cerebral oscillatory changes that occur during language tasks and actual language ability and therefore measured cerebral oscillatory changes using MEG, which directly reflects neural electrical activity.

The present study aimed to investigate the relationship between a clinical verbal memory index and language-related brain activity as detected by MEG. In healthy adults, we used MEG to measure ERS/ERD at θ (3–8 Hz), α (8–13 Hz), β (13–25 Hz), low γ (25–50 Hz) and high γ (50–100 Hz) frequency bands during a verb generation task. Then, we examined the correlation between spatiotemporal power in the brain and verbal memory score in Wechsler Memory Scale-revised (WMS-R), a general memory test.

## Results

### Spatiotemporal distribution of cerebral oscillatory changes

**Figure 1:**
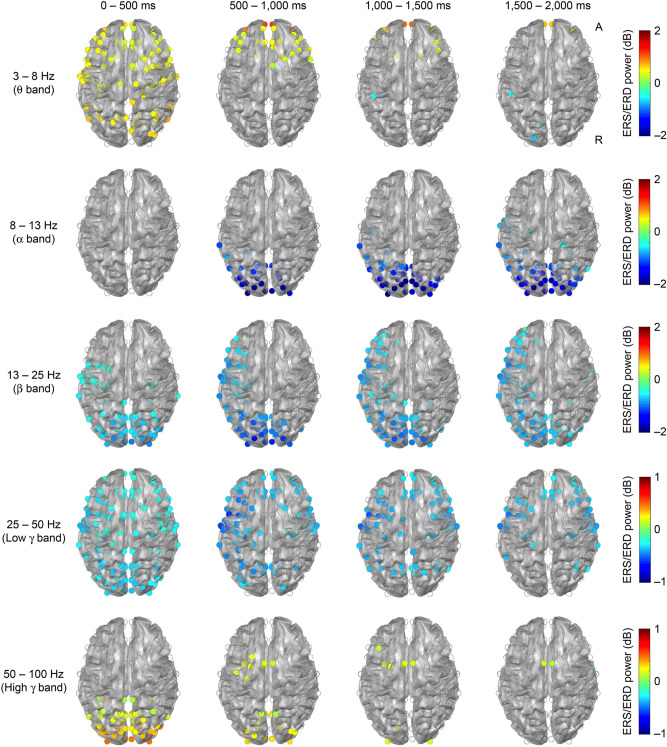
Spatiotemporal distribution of ERS/ERD. The average value of the spectral power of ERS/ERD derived from 160 points of virtual sensor in 36 subjects is shown. The baseline is set as − 500–0 ms. Only the power values for co-ordinates that were significant in the Wilcoxon signed-rank test (P < 0.001, Bonferroni correction) are shown. From the uppermost row, frequency bands shown are θ, α, β, low γ, and high γ band. There are four time sections: from leftmost column, these are 0–500, 500–1,000, 1,000–1,500 and 1,500–2,000 ms after presentation of the task. A positive value of spectral power indicates ERS, and a negative value indicates ERD. ‘A’ indicates anterior and ‘R’ indicates right.

Brain magnetic field activity was detected in 36 healthy subjects while a verb generation task was administered. The spatiotemporal distribution of ERS/ERD signals in the θ, α, β low γ and high γ frequency bands are shown (Fig. 1). Group analysis was performed based on the results of cerebral oscillatory changes in all subjects. In the θ band, ERS was widely observed in the bilateral frontal region and the parieto-occipital region at 0–500 ms after the task presentation, and was gradually localised to the bilateral straight gyrus after the 500–1,000 ms. After the 1,000–1,500 ms, ERD was found in the left postcentral and occipital region. In the α band, ERD was observed from the bilateral occipital region into the left central region at 500–1,000 ms after the task presentation and continued during the 1,500–2,000 ms interval. In the β band, ERD was observed from the bilateral occipital region into the left frontal region at 0–500 ms after the task presentation and the ERD widespread in the left hemisphere after the 500–1,000 ms. In the low γ band, ERD was more widespread at 0–500 ms after the presentation of the task. A higher power signal was recognised in the left frontal region during the 500–1,000 and 1,000–1,500 ms interval. At 1,500–2000 ms, ERD localised from the bilateral central to the frontal region was observed. In the high γ band, ERS was observed in the bilateral occipital region at 0–500 ms after the task presentation. After 500–1,000 ms, ERS in the occipital region decreased, and ERS was partially observed from the left frontal to the medial region.

### Correlation between ERS/ERD power and verbal memory score

**Figure 2:**
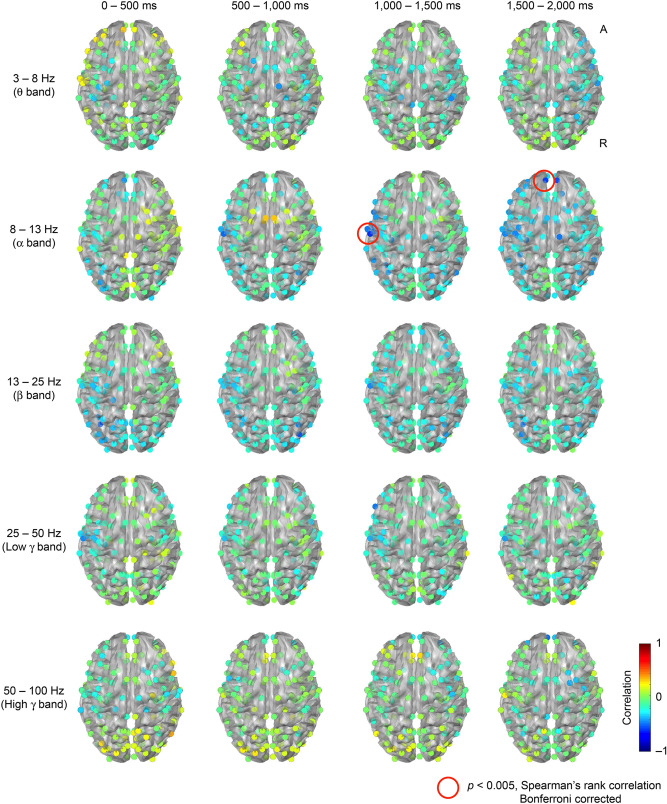
Correlation between ERS/ERD power and verbal memory. Correlation coefficients between the power value of ERS/ERD from virtual sensors at 160 points and the verbal memory score calculated by WMS-R are shown. From the uppermost row, frequency bands shown are θ, α, β, low γ, and high γ band. From the leftmost column, time sections shown are 0–500, 500–1,000, 1,000–1,500 and 1,500–2,000 ms after presentation of the task. Spearman's rank correlation coefficient was calculated and Bonferroni correction was applied to counteract multiple comparisons. Significant correlations (P < 0.05) were found at the co-ordinates circled in red.

**Figure 3:**
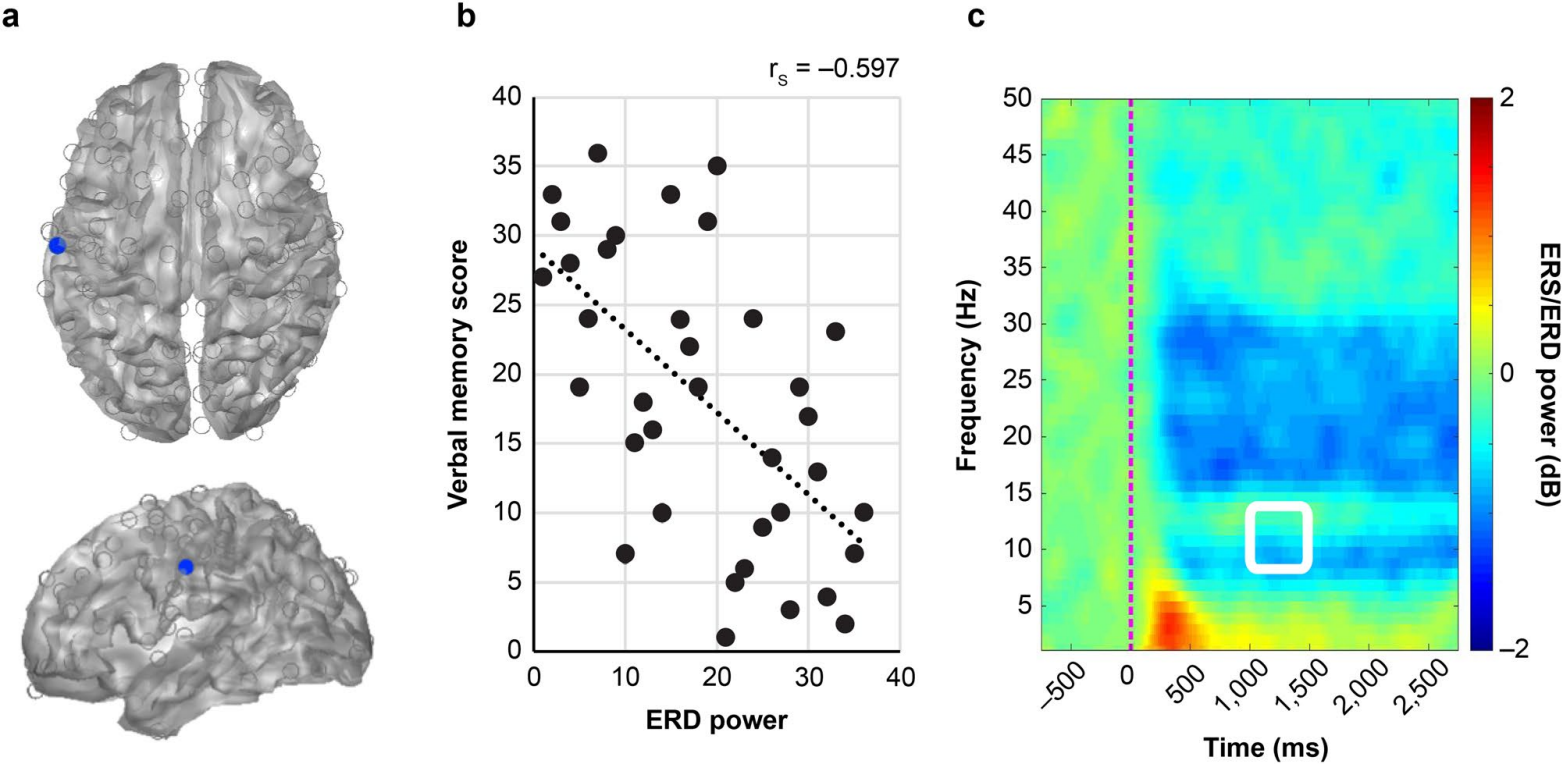
Co-ordinates of left central region and time–frequency spectrogram. (**a**) Co-ordinates of the left postcentral gyrus [MNI co-ordinates (− 59.8, − 16.3, 37.9)] that had a significant correlation at 1,000–1,500 ms in the α band after presentation of the task. (**b**) Correlation plot of the rank of absolute ERD power value and the rank of verbal memory score at the position of the left postcentral gyrus. Both ERD power value and verbal memory score were ranked in descending order. The dotted line represents a linear regression line, and r_s_ represents Spearman’s rank correlation coefficient. (**c**) Average time–frequency spectrogram in the postcentral gyrus from all subjects. The horizontal axis represents time, and the red dotted line represents the time at which the task was presented (0 ms). The vertical axis indicates frequency ranging from 1 to 50 Hz. The colour map shows the power spectrum of ERS/ERD. The area surrounded by a white square indicates area of 1,000–1,500 ms in the α band.

**Figure 4:**
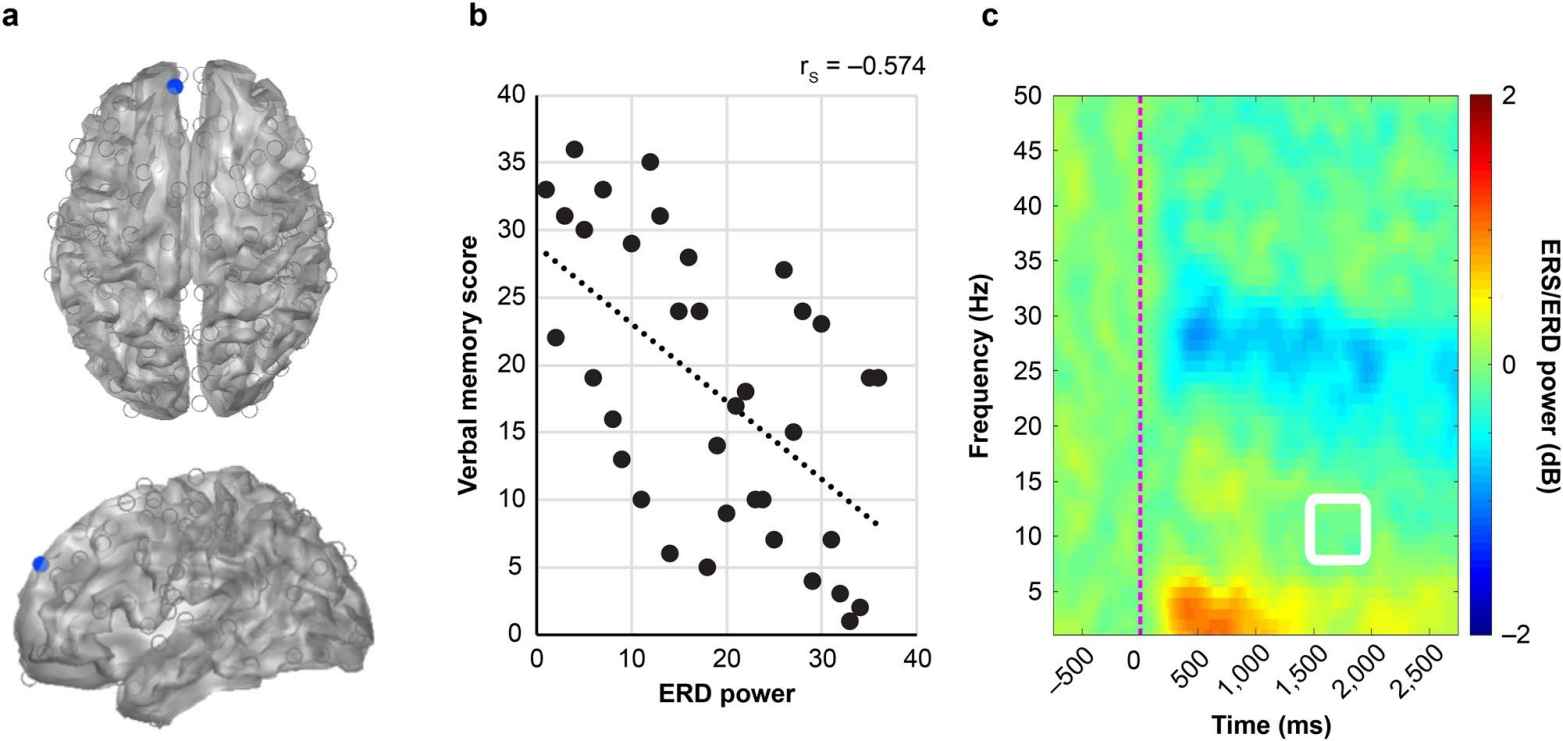
Co-ordinates of left medial superior frontal gyrus and time–frequency spectrogram. (**a**) The co-ordinates of the left medial superior frontal gyrus [MNI co-ordinates (− 7.3, 52.9, 30.9)] where a significant correlation was detected in the α band 1,500–2000 ms after task presentation. (**b**) Correlation plot of the rank of absolute ERD power value and the rank of verbal memory score at the position of left medial superior frontal gyrus. Both ERD power value and verbal memory score were ranked in descending order. The dotted line represents linear regression, and r_s_ represents Spearman's rank correlation coefficient. (**c**) Average time–frequency spectrogram in left medial superior frontal gyrus. The horizontal axis represents time, and the red dotted line represents the time at which the task was presented (0 ms). The vertical axis indicates the frequency ranging from 1 to 50 Hz. The colour map shows the power spectrum of ERS/ERD. The area surrounded by a white square indicates area of 1,500–2,000 ms in the α band.

Verbal memory scores were calculated from the WMS-R for each subject, and correlation coefficients were determined in conjunction with the spatiotemporal distribution of ERS/ERD power (Fig. 2). A significant negative correlation between verbal memory score and ERD power was observed in the left postcentral gyrus [MNI co-ordinates: (− 59.8, − 16.3, 37.9)] (Fig. 3a) at 1,000–1,500 ms in the α band (r_s_ =  − 0.597, P = 0.019) (Fig. 3b). A significant negative correlation was also observed in the left medial superior frontal gyrus [MNI co-ordinates (− 7.3, 52.9, 30.9)] (Fig. 4a) (r_s_ =  − 0.574, P = 0.041) at 1,500–2,000 ms in the α band (Fig. 4b).

In a time–frequency map of subject averages at each co-ordinate, ERDs of 15–30 Hz (Fig. 3c) were mainly observed in the left postcentral gyrus, while those of 25–30 Hz (Fig. 4c) were observed in the left medial superior frontal gyrus. Cerebral oscillatory changes in the β band, but not in the α band, tended to be commonly shared by all subjects.

## Discussion

In this study, we obtained ERS/ERD power measurements that correlate to brain activity during language tasks in 160 cortical regions. Then, we examined characteristics of spatiotemporal distribution of ERS/ERD powers and the correlation between the actual verbal memory score. During the verb generation task, differences in spatial distribution were revealed in five frequency bands—θ, α, β, low γ and high γ. We found that ERD was dominant in bilateral occipital regions in the α band from bilateral occipital regions extending to the left frontal region in the β band and in left frontal region in the low γ band. Goto et al. measured event-related changes using MEG in young healthy subjects during a silent reading task and observed ERD from the left occipital region extending to the temporal region in the α band, in the left central region in the β band and in the left frontal region in the low γ band^15^. Our previous study revealed that ERD in the low γ band is localised in the left middle frontal gyrus in healthy elderly subjects^14^. The findings of these previous studies agree with the results of the present study; this suggests that the spatial distribution of ERD may be independent of age. However, further research is required to verify this.

In the θ band, ERS was observed in a wide area from the frontal region to the occipital region immediately after the task presentation and then localised to the frontal region. In a previous study, ERS in the θ band was observed in the occipital region at 0–200 ms after the word presentation and then it was localised in the frontal region at 200–400 ms^15^. The θ-band ERS in the occipital region was observed preceding ERDs in the α, β, and low γ band; this ERS is the priming of the sustained ERDs. ERS in the frontal region is most probably associated with attention and verbal working memory load. In the present study, it is considered that ERS for priming and ERS related to attention and working memory are mixed in the 0–500 ms after the task presentation.

The high γ-band ERS recorded by electrocorticograms (ECoG) was reported to better reflect functional localisation and should therefore be a reliable marker of cortical activation^23,24^. Using MEG, Goto et al. reported that high-γ band ERS was observed in bilateral occipital regions^15^. In addition, Hashimoto et al. reported that ERS was observed in the left frontal region as well as the bilateral occipital region during verb generation task^25^. The findings of these previous studies agree with those of the present study. The ERS in the occipital region may represent neural activity in response to visual stimulation, and the ERS in the frontal region may represent neural activity in verbal processes.

Next, we found that α-band ERD power in the left postcentral gyrus and the left medial superior frontal gyrus was significantly negatively correlated with verbal memory score. Furthermore, in the left central region, the negative correlation coefficient approached similar values at peripheral sites such as precentral gyrus, inferior frontal gyrus and insula, although they were not significant (see Supplementary Fig. S1 online). However, these regions differ from the occipital region where ERD was detected as a commonality between all subjects by group analysis. In addition, the correlation between the power of ERS/ERD in five frequency bands and the visual memory score obtained from WMS-R was also examined, but there was no significant correlation (see Supplementary Fig. S2 online). These results suggest that verbal memory abilities may be related to α-band ERD fluctuations in the left central region and medial superior frontal gyrus.

It has been reported that oscillations in the α band is associated with cognitive function. Studies using EEG have shown that the higher the frequency in the α band of the resting state, the higher the cognitive function—information processing speed and memory in particular^26^. In addition, Bartsch et al. revealed that during a mental image language task that α-band oscillation increased in the occipital-parietal region when subjects were presented meaningful words, and this activity played an important role in the cognitive process of generating metaphorical language^27^. In a study using MEG, Van der Meer et al. revealed that multiple sclerosis patients with impaired cognitive function have increased spectral power in the α band at resting state in the occipital, parietal and temporal regions, and these powers were related to decreased cognitive function, especially information processing speed^28^. It may be considered that the ERD power in the α band during the processing of the language task in the present study also represented the cognitive abilities of each subject and showed a negative correlation with verbal memory. In the above studies, a relationship with cognitive function was found mainly in the occipital and parietal regions; however, in the present study, significant correlations were found in the left central region and the superior frontal region.

The central region of the brain is associated with verbal working memory. In ECoG study, Kambara et al. reported that inferior-precentral high-γ activity was augmented during the auditory naming task for patients aged 6–44^29^. In addition, this high-γ augmentation positively correlated with patient age. This result suggests that the performance of working memory from preschool to young adulthood was improved. In the study using fibre tractography, language-related sites of the temporal lobe were far more likely to be directly connected to the inferior precentral gyrus through the arcuate fasciculus; it was revealed that the central region plays an essential role in the language process neuroanatomically^30^. These studies are ECoG studies using auditory tasks and cannot be directly compared with our study. However, it is possible that the left central region was activated by maintaining the working memory in the process of generating a verb from word presentation. Moreover, it is considered that the strength of the activation is related to the performance of the working memory. Also, the central region of the brain is widely accepted as the oral movement and sensory control region^31^. Therefore, it is conceivable that the central region is involved in internal utterance of word or verb against the presented task. One-third of the presented words (34 out of 100) in this study were words that could be associated with the verbs ‘eat’ and ‘drink’. It has been reported that reading verbs related to body parts activates not only the Broca’s area, which controls motor speech, but also the primary motor area corresponding to that body part^32^. In this study, since subjects often associated oral movements such as eating and drinking after being presented words, it is possible that the sensorimotor cortex of the mouth was activated. At that time, the task load when generating verbs was small for individuals with high verbal memory, and the power change of ERD in the α band might be small.

The left medial superior frontal gyrus is an area called the dorsomedial prefrontal cortex (DMPFC), which has been known as an area that controls movement, attention, motivation and psychological processes^33^. It has been reported that the DMPFC on the left side is activated during metaphorical language generation^34^. Therefore, the DMPFC is considered to be an area related to creativity in language. In our study, brain activity negatively correlated with verbal memory was delayed from word presentation by 1,500–2,000 ms. This suggests that the DMPFC is activated in the process of recalling a verb after reading the word silently. However, as the functions related to the language process of DMPFC are often unclear, further studies are required to improve understanding of the functions associated with the DMPFC.

In processing language, the two brain regions described above demonstrate ERD in the α band, which plays a role in estimating the level of verbal memory. This suggests that using MEG to measure the ERD power in the α band may be used as a neurophysiological biomarker for estimating verbal memory.

There are some limitations to the present study. First, the correlation between the ERD in the α band and the verbal memory score was not statistically significant in most of the left central region, which may indicate that our study was insufficient. We believe that it is possible to show statistically significant correlations using other linguistic tasks and by conducting larger cohort studies. Furthermore, by considering not only WMS-R but also other indicators of language ability, the relationship between ERD in the α band and language ability will become clearer. Secondly, memory deteriorates with age and the raw scores obtained by WMS-R also decrease in the elderly. This study only considers young subjects, so it is unclear whether a similar correlation will be obtained in older subjects.

In conclusion, we found a significant negative correlation between ERD power in the α band in the left central region and left medial superior frontal gyrus and verbal memory score. This result indicates that people with lower linguistic memory have higher α-band ERD power and that α-band ERD power in the left hemisphere can be a neurophysiological biomarker representing verbal memory. We believe that this may contribute to the future development of a verbal memory test using MEG. At present, standard memory evaluation is conducted using neuropsychological tests such as WMS-R. However, the test load is heavy for patients with impaired cognitive function and some patients cannot complete the test. However, the language task used in this study is relatively simple and the duration of the test is approximately 10 min, thereby presenting a very small burden compared with that observed with current neuropsychological tests. In the future, we may even use MEG to evaluate patients whose cognitive function has deteriorated with this same language task. We will also compare non-WMS-R scores such as the Mini Mental State Examination. In this way, we aim to develop a language memory test using MEG.

## Methods

### Participants

The inclusion criteria consisted of healthy Japanese aged between 20 and 30 years old. The exclusion criteria consisted of: (i) left-handedness judged by the Edinburgh Handedness Inventory^35^, (ii) primary language other than Japanese, (iii) history of neurophysiological abnormalities. Thirty-six participants satisfying the inclusion and exclusion criteria were studied [mean age: 24.4; SD: 2.5 years]. Of these, 13 were men and 23 were women. After explaining the purpose of the study and possible outcome, informed consent was obtained from all subjects in writing before the MEG recordings. This study has been approved by the Ethics Review Committee of Osaka University (Approval number: 19035). We confirmed that all methods were performed in accordance with the relevant guidelines and regulations.

### MEG verbal task

**Figure 5:**
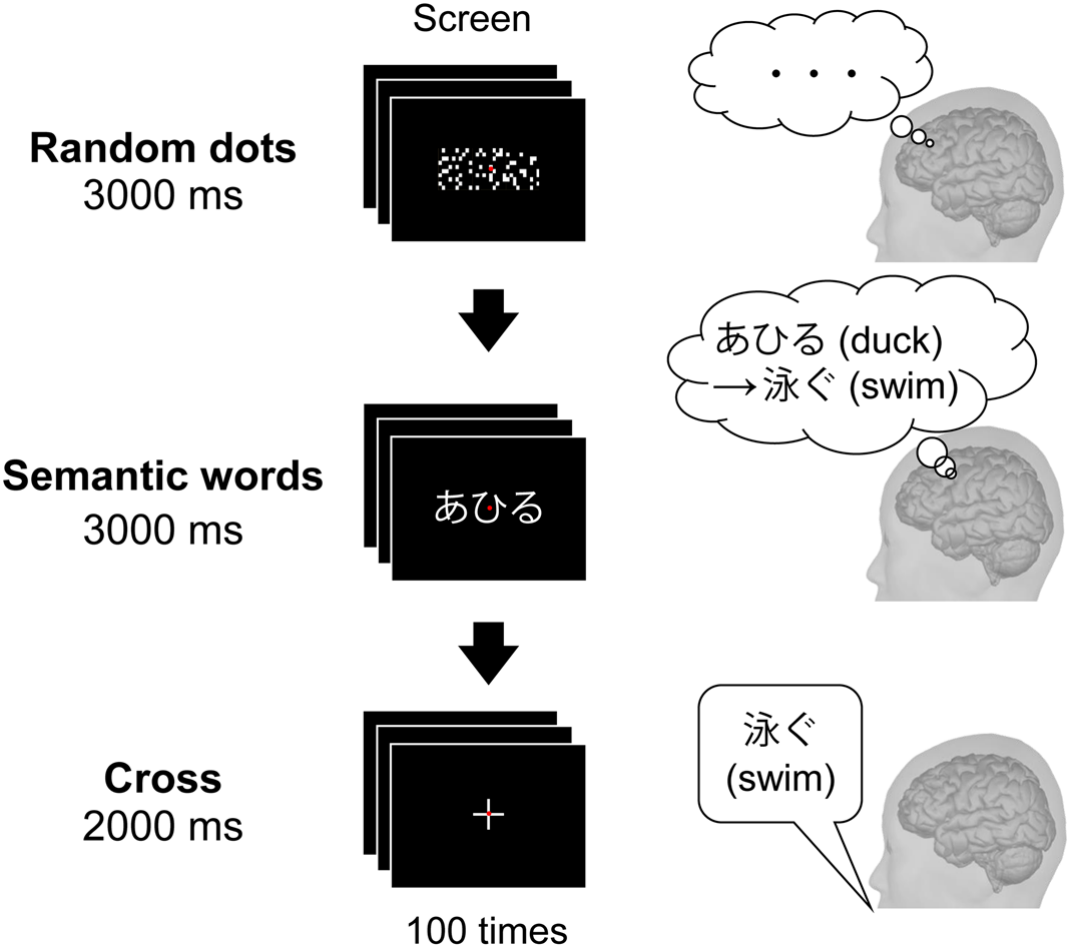
Flow of verb generation task. A screen was placed in front of each subjects’ eyes while in the MEG device. A random dot image was presented on the screen for 3,000 ms. The subjects were instructed to look at the screen without thinking during that period. Then, a meaningful Japanese word consisting of three characters of hiragana or katakana was presented for 3,000 ms. Subjects were instructed to silently read the word once upon presentation and recall verbs associated with that word. After that, a cross appeared for 2 s. The subject was instructed to utter the verb that he/she recalled immediately after the appearance of the cross. This sequence represents one trial, and 100 trials were performed.

Each participant performed a verb generation task during MEG measurement. Japanese words consisting of three morae of hiragana or katakana were presented as visual stimulation (Fig. 5). These words were selected from the NTT database series ‘Japanese Vocabulary Characteristics’^36^. First, a random dot image was presented for 3 s as a control stimulus and then a word was presented for 3 s. Participants were instructed to read the word silently once as soon as it was presented and then to generate only one verb associated with that word. After presenting the word for 3 s, a cross (+) was presented for 2 s. When the cross was presented, the participants spoke only the verb he or she generated. Then it was confirmed if the verb related to the original word. A total of 100 words were presented. A red circle was placed at the centre of all images and used as a fixation point.

### MEG measurement

We measured brain magnetic field activity with 160-channel whole-head magnetoencephalography (MEG Vision NEO; Ricoh Company, Ltd., Yokohama, Kanagawa, Japan). Subjects lay on a bed on their back with their head centred. A screen for visual stimulation was installed at a distance of 325 mm in front of the subject. Visual stimulation was given by a liquid crystal projector operated by visual presentation software (Presentation; Neurobehavioral Systems, Inc., Berkeley, CA, USA). Subjects were instructed not to move their heads or not to exert any unnecessary force during the measurement. We measured brain anatomical images using a 3 T-MRI apparatus (Sigma Excite HDxt 3.0 T; GE Healthcare Life Sciences, Amersham, Buckinghamshire, England). To integrate the individual MEG and MRI data, we scanned the facial surface using a 3D scanning device (FastSCAN Cobra; Polhemus, Colchester, VT, USA). To measure the head position during MEG measurement, a total of five marker coils were attached before MEG measurement (left and right pre-auricular areas and three forehead areas). The sampling frequency of MEG data was 1,000 Hz, and a low pass filter of 200 Hz was applied. After measuring the data, we applied a high pass filter of 1 Hz to remove DC offset and a 60 Hz notch filter to remove line noise. Blinking artefacts were removed by using a signal-space projection built into a software program [Brainstorm; (https://neuroimage.usc.edu/brainstorm)] that runs on MATLAB^37^.

### MEG analysis

A total of 160 virtual sensors were set in the cerebral cortex. Then, the power of ERS/ERD for each frequency band was calculated using time–frequency analysis for an estimated signal of each virtual sensor. The total number of virtual sensor co-ordinates was 160 points (see Supplemental Table S1 online). First, a SPM12 (https://www.fil.ion.ucl.ac.uk/spm/) file called cortex_5124.surf.gii that runs on MATLAB was down sampled. After extracting 80 points in the left hemisphere, those of the right hemisphere that were symmetrical to 80 points in the left hemisphere were extracted. BRAM toolbox (BRAM; Ricoh Company, Ltd., Yokohama, Kanagawa, Japan) was used for signal estimation at each virtual sensor^38^. Next, a time–frequency analysis was conducted for the estimated signals from 160 virtual sensors of each subject. Time–frequency analysis was conducted using software called EEGLAB (https://www.sccn.ucsd.edu/eeglab) running on MATLAB^39^. In time–frequency analysis, spectral power compared to the baseline was calculated as a two-dimensional map with frequency on the vertical axis and time on the horizontal axis. The signal interval used for this analysis was set between 1,000 ms before and 3,000 ms after the stimulus presentation. For individual data, epochs contaminated with artefacts such as EMG were excluded from the analysis. Epochs from each individual were analysed by short-time Fourier transform using FFT. The baseline was set from 500 ms before stimulus presentation to 0 ms, and the frequency band used for analysis was between 1 and 100 Hz. Average spectral power was calculated based on the results of time–frequency analysis. Frequency bands used for this analysis were 3–8, 8–13, 13–25, 25–50 and 50–100 Hz. Time intervals used were four intervals of 0–500, 500–1,000, 1,000–1,500, 1,500–2000 ms after the stimulus presentation.

### Correlation with verbal memory score

To examine the relationship between ERD and actual verbal memory during language tasks, a Japanese version of WMS-R was conducted on the same day as MEG measurement. WMS-R is a memory test consisting of five evaluation items: verbal memory, visual memory, general memory, attention/concentration and delayed recall^40^. The higher the scores, the higher is the performance. Among these five items, this study focused on verbal memory. The spectral power value of ERD in each frequency band and time interval in each virtual sensor was calculated.

### Statistical analysis

The average value at each frequency and time interval was calculated and significant co-ordinates identified by the Wilcoxon signed-rank test (P < 0.001, Bonferroni correction) were displayed on a colour map on the virtual sensor co-ordinates of the standard brain co-ordinate system. The correlation coefficient between those power values and verbal memory score was calculated using Spearman’s rank correlation coefficient. The co-ordinates for which a statistically significant correlation (P < 0.05, Bonferroni correction) coefficient was given were displayed as a colour map.

## Supplementary information

Supplementary Information

## Data Availability

The datasets during and/or analysed during the current study available from the corresponding author on reasonable request.
