# Supplementary material for: Left hemispheric α band cerebral oscillatory changes correlate with verbal memory
Source: Sci Rep. 2020 Sep 14;10:14993. doi: 10.1038/s41598-020-72087-3 (PMC7490359; doi:10.1038/s41598-020-72087-3)
Supplement: Supplementary file 1 — Supplementary Information [file 41598_2020_72087_MOESM1_ESM.pdf]

Supplementary Information for
Left hemispheric α band cerebral oscillatory changes correlate with verbal memory

Toshihiko Araki[1,2], Yoshiyuki Watanabe[3], Masayuki Hirata[4]

[1]Department of Medical Technology, Osaka University Hospital, Suita, Osaka, Japan
[2]Division of Health Sciences, Osaka University Graduate School of Medicine, Suita, Osaka, Japan
[3]Department of Radiology, Shiga University of Medical Science, Otsu, Shiga, Japan
[4]Department of Neurological Diagnosis and Restoration, Osaka University Graduate School of Medicine, Suita, Osaka, Japan

Contents:

Supplementary Figure S1, S2
Supplementary Table S1

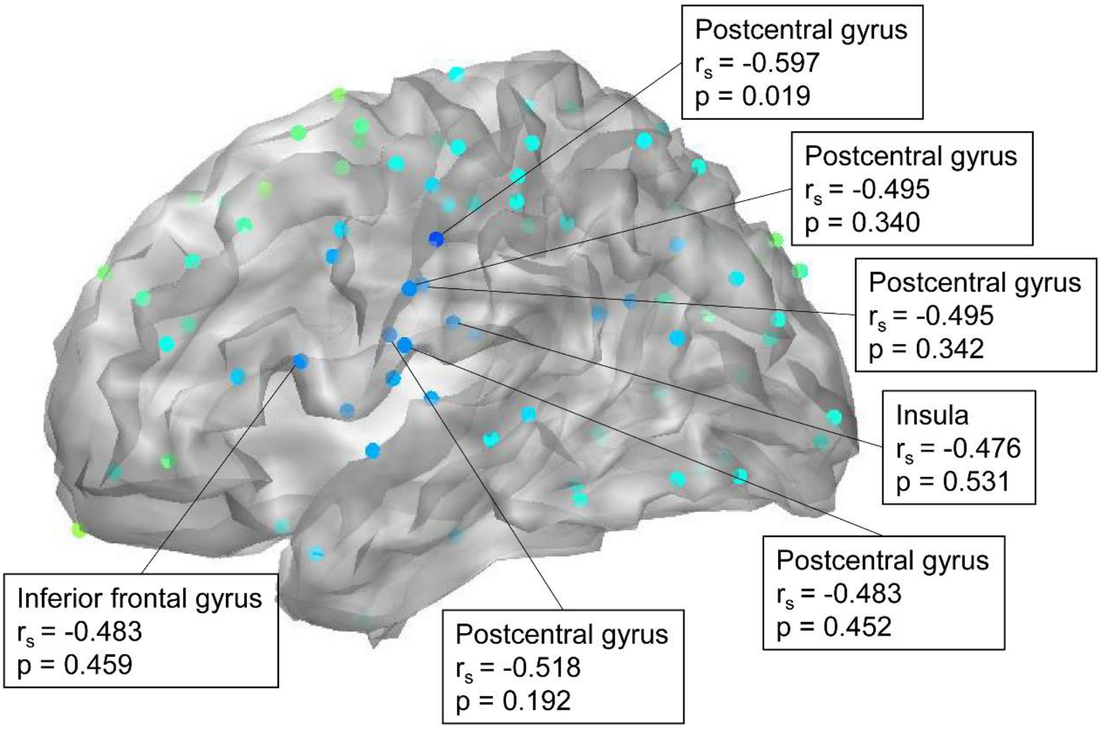

Supplementary Figure S1. Correlation coefficients between α band event-related desynchronisation power at 1000–1500 ms and verbal memory score

Co-ordinates with a negative correlation coefficient of 0.47 or more in the left central region are shown. Brain co-ordinates are accompanied by names of the brain regions, Spearman's rank correlation coefficients and P values.

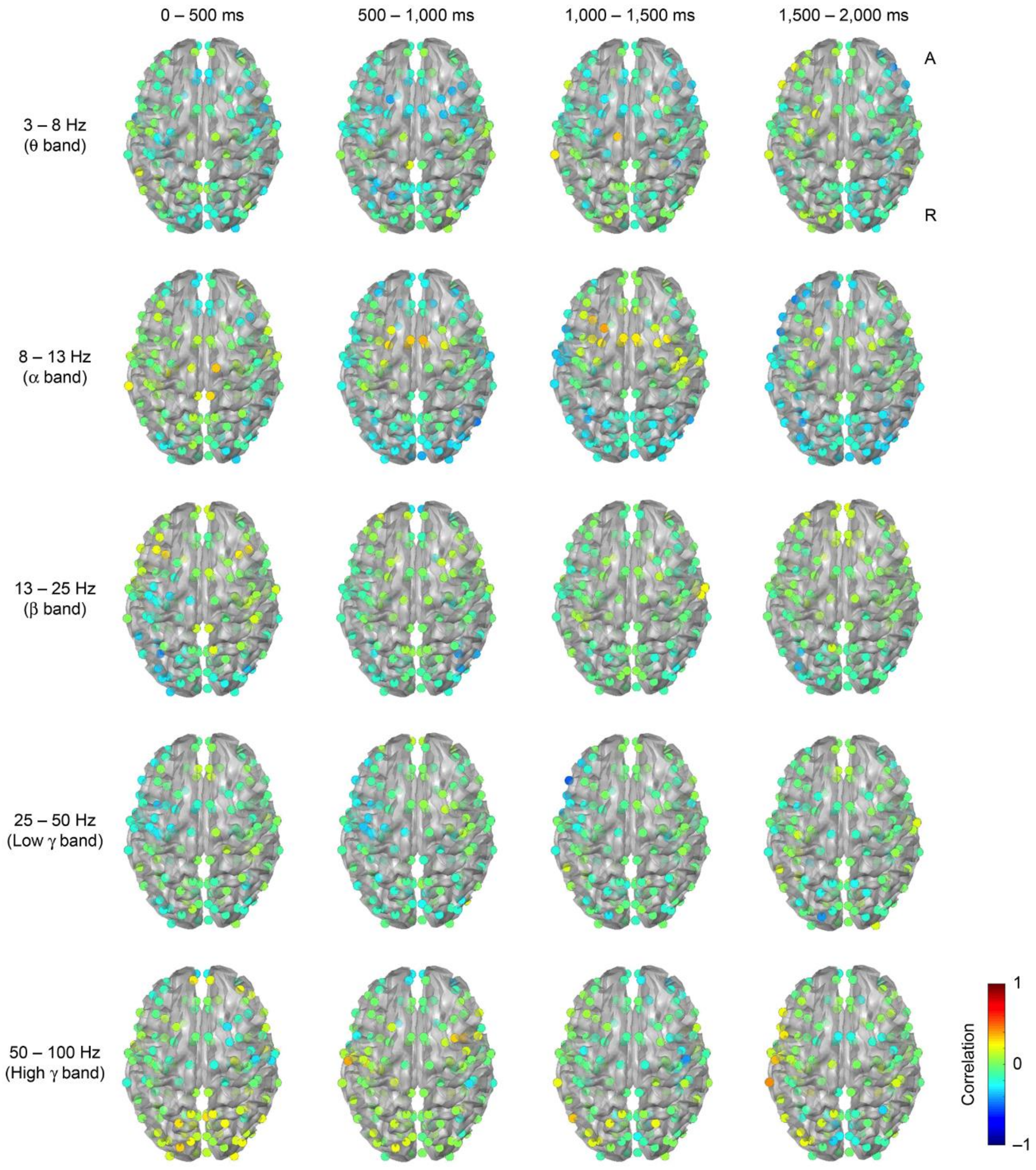

Supplementary Figure S2. Correlation between event-related synchronisation/desynchronisation (ERS/ERD) power and visual memory

Correlation coefficients between the power value of ERS/ERD from virtual sensors at 160 points and the visual memory score calculated by WMS-R are shown. From the uppermost row, frequency band shown are θ, α, β, low γ, and high γ band. From the leftmost column, time sections shown are 0–500, 500–1000, 1000–1500 and 1500–2000 ms after presentation of the task. Spearman's rank correlation coefficient was calculated and Bonferroni correction was applied to counteract multiple comparisons. There were no significant correlations ($P < 0.05$).

Supplementary Table S1. MNI co-ordinates of 160 virtual sensors

No	MNI co-ordinates			Hemisphere	Brain region
	X	Y	Z		
1	−27.7	−98.8	0.9	Left	Middle occipital gyrus
2	−4.4	−95.9	−4.4	Left	Calcarine
3	−19.0	−91.4	31.4	Left	Superior occipital gyrus
4	−9.6	−86.3	37.8	Left	Cuneus
5	−34.8	−87.2	21.2	Left	Middle occipital gyrus
6	−29.3	−85.5	17.4	Left	Middle occipital gyrus
7	−4.3	−82.0	33.6	Left	Cuneus
8	−14.6	−79.3	9.3	Left	Calcarine
9	−37.9	−78.5	29.7	Left	Middle occipital gyrus
10	−42.3	−79.2	−11.4	Left	Inferior occipital gyrus
11	−13.8	−75.8	−7.8	Left	Lingual gyrus
12	−11.8	−72.5	22.3	Left	Cuneus
13	−7.7	−72.4	37.9	Left	Precuneus
14	−19.9	−70.6	53.0	Left	Superior parietal lobule
15	−21.2	−71.1	−10.5	Left	Lingual gyrus
16	−4.1	−65.0	44.5	Left	Precuneus
17	−36.7	−66.1	37.0	Left	Angular gyrus
18	−9.3	−62.6	60.8	Left	Precuneus
19	−51.6	−66.4	17.4	Left	Middle temporal gyrus
20	−50.3	−66.3	−12.1	Left	Inferior occipital gyrus
21	−14.5	−63.6	25.2	Left	Cuneus
22	−4.6	−62.6	6.0	Left	Lingual gyrus
23	−33.1	−59.4	58.4	Left	Superior parietal lobule
24	−39.5	−56.7	24.9	Left	Angular gyrus
25	−55.1	−50.2	22.4	Left	Middle temporal gyrus
26	−5.5	−44.0	65.2	Left	Precuneus
27	−12.0	−49.7	−2.6	Left	Lingual gyrus
28	−56.4	−46.1	−16.4	Left	Inferior temporal gyrus
29	−40.2	−43.3	41.4	Left	Inferior parietal lobule
30	−29.7	−45.0	−13.1	Left	Fusiform gyrus
31	−44.8	−36.1	57.9	Left	Postcentral gyrus

32	−38.6	−35.2	40.8	Left	Inferior parietal lobule
33	−48.5	−33.1	51.1	Left	Postcentral gyrus
34	−20.0	−34.9	65.5	Left	Postcentral gyrus
35	−64.9	−35.5	1.4	Left	Middle temporal gyrus
36	−44.6	−32.9	45.8	Left	Postcentral gyrus
37	−53.9	−27.6	−3.6	Left	Middle temporal gyrus
38	−37.9	−23.7	45.3	Left	Postcentral gyrus
39	−39.3	−20.6	57.0	Left	Precentral gyrus
40	−32.1	−24.1	18.4	Left	Insula
41	−10.6	−20.1	72.2	Left	Paracentral lobule
42	−36.8	−19.6	20.5	Left	Insula
43	−28.2	−16.5	58.3	Left	Precentral gyrus
44	−44.1	−18.6	45.1	Left	Postcentral gyrus
45	−24.7	−20.1	−24.0	Left	Parahippocampal gyrus
46	−59.8	−16.3	37.9	Left	Postcentral gyrus
47	−48.9	−15.2	4.8	Left	Superior temporal gyrus
48	−46.7	−15.5	49.0	Left	Postcentral gyrus
49	−55.0	−13.3	28.4	Left	Postcentral gyrus
50	−62.9	−10.8	27.6	Left	Postcentral gyrus
51	−63.3	−9.8	15.8	Left	Postcentral gyrus
52	−42.1	−7.8	53.6	Left	Precentral gyrus
53	−51.7	−7.2	9.0	Left	Rolandic operculum
54	−54.2	−6.5	18.0	Left	Postcentral gyrus
55	−58.0	−3.1	−6.1	Left	Superior temporal gyrus
56	−24.1	−0.4	61.5	Left	Middle frontal gyrus
57	−22.0	12.6	60.1	Left	Middle frontal gyrus
58	−7.6	0.1	58.2	Left	Supplementary motor area
59	−5.4	4.3	67.8	Left	Supplementary motor area
60	−49.5	2.4	2.1	Left	Rolandic operculum
61	−26.4	−1.3	−41.7	Left	Inferior temporal gyrus
62	−39.8	3.5	40.0	Left	Precentral gyrus
63	−52.2	5.2	34.4	Left	Precentral gyrus
64	−46.1	8.9	−27.4	Left	Middle temporal pole
65	−54.9	11.9	12.6	Left	Opercular part of inferior frontal gyrus

66	−16.5	16.0	−22.0	Left	Orbital part of inferior frontal gyrus
67	−53.1	24.9	9.3	Left	Triangular part of the inferior frontal gyrus
68	−39.7	23.8	40.8	Left	Middle frontal gyrus
69	−6.6	28.0	45.5	Left	Medial superior frontal gyrus
70	−5.0	34.8	46.3	Left	Medial superior frontal gyrus
71	−37.1	34.5	33.3	Left	Middle frontal gyrus
72	−14.0	35.6	20.3	Left	Anterior cingulum
73	−40.5	39.5	−8.1	Left	Orbital part of inferior frontal gyrus
74	−42.2	40.0	16.2	Left	Middle frontal gyrus
75	−32.8	44.9	25.6	Left	Middle frontal gyrus
76	−32.3	3.5	52.8	Left	Middle frontal gyrus
77	−31.8	19.5	48.3	Left	Middle frontal gyrus
78	−30.8	51.0	−10.4	Left	Orbital part of inferior frontal gyrus
79	−7.3	52.9	30.9	Left	Medial superior frontal gyrus
80	−5.1	58.4	−22.5	Left	Straight gyrus
81	27.7	−98.8	0.9	Right	Middle occipital gyrus
82	4.4	−95.9	−4.4	Right	Calcarine
83	19.0	−91.4	31.4	Right	Superior occipital gyrus
84	9.6	−86.3	37.8	Right	Cuneus
85	34.8	−87.2	21.2	Right	Middle occipital gyrus
86	29.3	−85.5	17.4	Right	Middle occipital gyrus
87	4.3	−82.0	33.6	Right	Cuneus
88	14.6	−79.3	9.3	Right	Calcarine
89	37.9	−78.5	29.7	Right	Middle occipital gyrus
90	42.3	−79.2	−11.4	Right	Inferior occipital gyrus
91	13.8	−75.8	−7.8	Right	Lingual gyrus
92	11.8	−72.5	22.3	Right	Cuneus
93	7.7	−72.4	37.9	Right	Precuneus
94	19.9	−70.6	53.0	Right	Superior parietal lobule
95	21.2	−71.1	−10.5	Right	Lingual gyrus
96	4.1	−65.0	44.5	Right	Precuneus
97	36.7	−66.1	37.0	Right	Middle occipital gyrus
98	9.3	−62.6	60.8	Right	Precuneus
99	51.6	−66.4	17.4	Right	Middle temporal gyrus

100	50.3	−66.3	−12.1	Right	Inferior temporal gyrus
101	14.5	−63.6	25.2	Right	Precuneus
102	4.6	−62.6	6.0	Right	Lingual gyrus
103	33.1	−59.4	58.4	Right	Superior parietal lobule
104	39.5	−56.7	24.9	Right	Angular gyrus
105	55.1	−50.2	22.4	Right	Superior temporal gyrus
106	5.5	−44.0	65.2	Right	Paracentral lobule
107	12.0	−49.7	−2.6	Right	Lingual gyrus
108	56.4	−46.1	−16.4	Right	Inferior temporal gyrus
109	40.2	−43.3	41.4	Right	Inferior parietal lobule
110	29.7	−45.0	−13.1	Right	Fusiform gyrus
111	44.8	−36.1	57.9	Right	Postcentral gyrus
112	38.6	−35.2	40.8	Right	Supramarginal gyrus
113	48.5	−33.1	51.1	Right	Inferior parietal lobule
114	20.0	−34.9	65.5	Right	Postcentral gyrus
115	64.9	−35.5	1.4	Right	Middle temporal gyrus
116	44.6	−32.9	45.8	Right	Postcentral gyrus
117	53.9	−27.6	−3.6	Right	Middle temporal gyrus
118	37.9	−23.7	45.3	Right	Postcentral gyrus
119	39.3	−20.6	57.0	Right	Precentral gyrus
120	32.1	−24.1	18.4	Right	Insula
121	10.6	−20.1	72.2	Right	Supplementary motor area
122	36.8	−19.6	20.5	Right	Rolandic operculum
123	28.2	−16.5	58.3	Right	Precentral gyrus
124	44.1	−18.6	45.1	Right	Precentral gyrus
125	24.7	−20.1	−24.0	Right	Parahippocampal gyrus
126	59.8	−16.3	37.9	Right	Postcentral gyrus
127	48.9	−15.2	4.8	Right	Heschl’s gyrus
128	46.7	−15.5	49.0	Right	Precentral gyrus
129	55.0	−13.3	28.4	Right	Supramarginal gyrus
130	62.9	−10.8	27.6	Right	Postcentral gyrus
131	63.3	−9.8	15.8	Right	Postcentral gyrus
132	42.1	−7.8	53.6	Right	Middle frontal gyrus
133	51.7	−7.2	9.0	Right	Heschl’s gyrus

134	54.2	−6.5	18.0	Right	Rolandic operculum
135	58.0	−3.1	−6.1	Right	Superior temporal gyrus
136	24.1	−0.4	61.5	Right	Superior frontal gyrus
137	22.0	12.6	60.1	Right	Superior frontal gyrus
138	7.6	0.1	58.2	Right	Supplementary motor area
139	5.4	4.3	67.8	Right	Supplementary motor area
140	49.5	2.4	2.1	Right	Rolandic operculum
141	26.4	−1.3	−41.7	Right	Fusiform gyrus
142	39.8	3.5	40.0	Right	Middle frontal gyrus
143	52.2	5.2	34.4	Right	Precentral gyrus
144	46.1	8.9	−27.4	Right	Middle temporal pole
145	54.9	11.9	12.6	Right	Opercular part of inferior frontal gyrus
146	16.5	16.0	−22.0	Right	Orbital part of inferior frontal gyrus
147	53.1	24.9	9.3	Right	Triangular part of the inferior frontal gyrus
148	39.7	23.8	40.8	Right	Middle frontal gyrus
149	6.6	28.0	45.5	Right	Medial superior frontal gyrus
150	5.0	34.8	46.3	Right	Medial superior frontal gyrus
151	37.1	34.5	33.3	Right	Middle frontal gyrus
152	14.0	35.6	20.3	Right	Anterior cingulum
153	40.5	39.5	−8.1	Right	Orbital part of inferior frontal gyrus
154	42.2	40.0	16.2	Right	Middle frontal gyrus
155	32.8	44.9	25.6	Right	Middle frontal gyrus
156	32.3	3.5	52.8	Right	Middle frontal gyrus
157	31.8	19.5	48.3	Right	Middle frontal gyrus
158	30.8	51.0	−10.4	Right	Orbital part of inferior frontal gyrus
159	7.3	52.9	30.9	Right	Medial superior frontal gyrus
160	5.1	58.4	−22.5	Right	Straight gyrus
